# Clinical Outcome of High-Risk Patients with Severe Aortic Stenosis and Reduced Left Ventricular Ejection Fraction Undergoing Medical Treatment or TAVI

**DOI:** 10.1371/journal.pone.0027556

**Published:** 2011-11-10

**Authors:** Thomas Pilgrim, Peter Wenaweser, Fabienne Meuli, Christoph Huber, Stefan Stortecky, Christian Seiler, Stephan Zbinden, Bernhard Meier, Thierry Carrel, Stephan Windecker

**Affiliations:** 1 Department of Cardiology, Swiss Cardiovascular Center, Bern University Hospital, Bern, Switzerland; 2 Department of Cardiovascular Surgery, Swiss Cardiovascular Center, Bern University Hospital, Bern, Switzerland; University of Modena and Reggio Emilia, Italy

## Abstract

**Introduction:**

Reduced left ventricular function in patients with severe symptomatic valvular aortic stenosis is associated with impaired clinical outcome in patients undergoing surgical aortic valve replacement (SAVR). Transcatheter Aortic Valve Implantation (TAVI) has been shown non-inferior to SAVR in high-risk patients with respect to mortality and may result in faster left ventricular recovery.

**Methods:**

We investigated clinical outcomes of high-risk patients with severe aortic stenosis undergoing medical treatment (n = 71) or TAVI (n = 256) stratified by left ventricular ejection fraction (LVEF) in a prospective single center registry.

**Results:**

Twenty-five patients (35%) among the medical cohort were found to have an LVEF≤30% (mean 26.7±4.1%) and 37 patients (14%) among the TAVI patients (mean 25.2±4.4%). Estimated peri-interventional risk as assessed by logistic EuroSCORE was significantly higher in patients with severely impaired LVEF as compared to patients with LVEF>30% (medical/TAVI 38.5±13.8%/40.6±16.4% versus medical/TAVI 22.5±10.8%/22.1±12.8%, p <0.001). In patients undergoing TAVI, there was no significant difference in the combined endpoint of death, myocardial infarction, major stroke, life-threatening bleeding, major access-site complications, valvular re-intervention, or renal failure at 30 days between the two groups (21.0% versus 27.0%, p = 0.40). After TAVI, patients with LVEF≤30% experienced a rapid improvement in LVEF (from 25±4% to 34±10% at discharge, p = 0.002) associated with improved NYHA functional class at 30 days (decrease ≥1 NYHA class in 95%). During long-term follow-up no difference in survival was observed in patients undergoing TAVI irrespective of baseline LVEF (p = 0.29), whereas there was a significantly higher mortality in medically treated patients with severely reduced LVEF (log rank p = 0.001).

**Conclusion:**

TAVI in patients with severely reduced left ventricular function may be performed safely and is associated with rapid recovery of systolic left ventricular function and heart failure symptoms.

## Introduction

Reduced left ventricular ejection fraction (LVEF) among patients with severe aortic stenosis importantly impacts prognosis in patients treated conservatively, and increases peri-operative risk in patients undergoing surgical aortic valve replacement (SAVR) [1]–[4]. Although mechanical relief of aortic outflow obstruction as accomplished by SAVR has been shown to compensate for the increased peri-operative risk in patients with decreased LVEF [5], severely impaired LVEF remains one of the principal reasons to defer SAVR [6]. Moreover, recovery of LVEF in response to SAVR remains variable and difficult to predict. Transcatheter Aortic Valve Implantation (TAVI) is a less invasive procedure, which is predominantly performed among patients previously managed by medical treatment. In patients not considered suitable candidates for SAVR, TAVI has been shown to reduce mortality and rehospitalization compared with a conservative strategy [7]. In addition, it has been suggested that TAVI is associated with favorable effects on LVEF recovery [8]. The safety and efficacy of TAVI in patients with reduced LVEF (≤30%) vis-à-vis a conservative strategy has not been resolved. Therefore, we investigated clinical outcomes of high-risk patients with severe aortic stenosis undergoing medical treatment or TAVI stratified by LVEF in a prospective single-center registry.

## Methods

### Patient Population

High-risk patients with symptomatic, severe aortic stenosis deemed at increased surgical risk have been consecutively included in a prospective single center registry initiated in July 2007. Inclusion criteria involved (1) symptomatic, severe aortic stenosis with an echocardiographic mean gradient >40 mmHg or a calculated aortic valve area <1 cm^2^; (2) age ≥80 years in the presence of a logistic EuroSCORE >15%. Patients <80 years of age were eligible if at least one of the following comorbid conditions was present: previous cardiac surgery, chronic obstructive pulmonary disease (forced expiratory volume during one second <1.0), severe pulmonary hypertension (≥60 mmHg), porcelain aorta, history of radiation therapy to the mediastinum, or frailty (BMI <18 kg/m^2^). Patients with severe aortic regurgitation due to degenerated aortic valve prosthesis were excluded.

### Ethics Statement

The study complies with the Declaration of Helsinki and was approved by the Ethics Committee of the University of Bern, Switzerland (www.kek-bern.ch). All subjects gave informed written consent.

### Evaluation and Treatment

After a comprehensive evaluation according to a standardized protocol including left and right heart catheterization, aortography, transthoracic (TTE) and transesophageal echocardiography (TEE), and CT angiography of the chest, abdomen and pelvis an interdisciplinary team of interventional cardiologists and cardiac surgeons reviewed the cases and formed a consensus on treatment allocation to medical treatment (1), SAVR (2) or TAVI (3) based on risk assessment, anatomical considerations and patient preference [9]. For the purpose of this analysis, we focused on patients allocated to medical treatment or TAVI. Allocation to medical treatment resulted from patient's refusal to undergo either SAVR or TAVI despite the recommendation for an intervention put forward by the heart team, comorbidities with poor prognosis, anatomical or technical reasons not allowing for a transcatheter approach in patients refusing to undergo SAVR, and exceedingly high estimated risk for intervention. Reasons for TAVI included refusal of SAVR, advanced age (>80 years) in the setting of a high surgical risk, or severe comorbidities. Patients with cross-over from medical treatment or TAVI to SAVR or from medical treatment to TAVI during the time of follow-up were excluded. Medical treatment encompassed percutaneous coronary intervention in case of significant coronary artery disease with limiting angina, as well as optimal medical treatment for comorbidities such as congestive heart failure, atrial fibrillation, and hypertension, and was not necessarily associated with an equally conservative strategy for treatment of non-cardiac disease manifestations. Conservative treatment did however not include balloon aortic valvuloplasty. Also, it was not used as a bridge to TAVI.

Latter was performed through a tranfemoral, transapical, or transsubclavian approach according to anatomical characteristics using either the Medtronic CoreValve Revalving system or the Edwards Sapien valve as previously described [10].

### Data collection

Patients treated medically were included into this registry at the time of the in-hospital evaluation for a potential intervention, whereas the date of the intervention was considered the time of inclusion among patients undergoing TAVI. Follow-up was performed regularly at 1, 6, and 12 months during a clinic visit or by means of a standardized telephone interview. Furthermore, all patients were contacted within two months of data freezing for the purpose of the present analysis. Hospital records and municipal civil registries were consulted to ascertain vital status. Medical records, discharge letters, and documentations of hospitalizations were systematically collected and all suspected events were adjudicated by an unblinded clinical event committee consisting of cardiac surgeons and interventional cardiologists.

### Definitions

Assessment of left ventricular ejection fraction at baseline was based on measurements from TTE using planimetry. All endpoint definitions were in accordance with the criteria suggested by the Valve Academic Research Consortium [11]. The definition of cardiovascular death involved any death due to a proximate cardiac cause or a death of unknown cause, as well as all procedure-related deaths and death caused by non-coronary vascular conditions such as cerebrovascular disease, pulmonary embolism, or other vascular disease. Peri-procedural myocardial infarction was determined as new ischemic symptoms or signs in the presence of elevated cardiac biomarkers (two or more post-procedure samples that were >6–8 hours apart with a 20% increase in the second sample and a peak value exceeding 10x the 99^th^ percentile upper reference limit (URL), or a peak value exceeding 5x the 99^th^ percentile URL with new pathological Q waves in at least two contiguous leads) within 72 hours after the index procedure. Major stroke encompassed a rapid onset of focal or global neurological deficit of ≥24 hours duration necessitating therapeutic intervention, or documentation of a new intracranial defect using MRI or CT-scan. The modified RIFLE (Risk, Injury, Failure, Loss, End-stage kidney disease) classification was used for the definition of kidney injury which was based upon changes in serum creatinine up to 72 hours after the procedure. Stage 1 was determined as an increase of serum creatinine to 150–200% (or an increase of ≥26.4 µmol/l), stage 2 required an increase of the baseline creatinine to 200–300%, and stage 3 was considered in case of an increase in creatinine of ≥300% with an acute increase of at least 44 µmol/l.

### Statistical Analysis

All analyses were performed using SPSS Statistics Version 17.0. Continuous variables are presented as mean ± standard deviation (SD) and were compared by means of a two-sided students T-test. Categorical data are expressed as frequency (percentages), and were compared using the chi-square and Fishers exact tests. Survival was estimated using the Kaplan Meier method. We performed an univariate analysis and included in addition to age and gender all variables with a p<0.1 into a cox multivariate regression model to adjust for potential confounders.

## Results

### Baseline Characteristics

**Figure 1:**
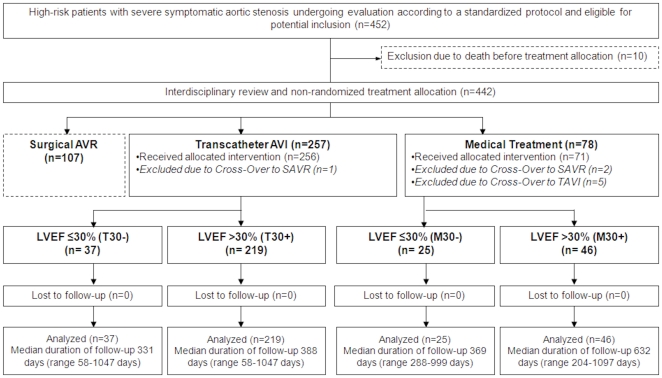
Patient flow according to CONSORT statement.

Among 452 patients with severe aortic stenosis at increased surgical risk, 10 patients died before treatment allocation, and 107 patients underwent SAVR leaving 335 patients allocated to medical treatment or TAVI as basis of the present study. After exclusion of two patients with cross-over from medical treatment to SAVR, one patient with cross-over from TAVI to SAVR, and five patients with cross-over from medical treatment to TAVI, 71 patients treated medically and 256 patients treated by TAVI remained for the purpose of the present analysis. The patient population was divided into four groups according to treatment strategy (medical treatment versus TAVI) and left ventricular ejection fraction (LVEF≤30% versus LVEF>30%). Severely impaired LVEF (≤30%) was observed in 25 (35%) patients treated medically (M30-), whereas 46 (65%) patients treated medically had normal or only moderately reduced LVEF (M30+). A total of 37 (14%) TAVI patients had severely diminished LVEF (T30-), whereas 219 (86%) TAVI patients showed normal or moderately reduced LVEF (T30+) (Figure 1). Baseline characteristics of the overall patient population undergoing medical treatment or TAVI stratified by ventricular function are summarized in Tables 1 and 2. The estimated peri-procedural risk was higher in the M30- group as compared to the M30+ group (logistic EuroSCORE 38.5±13.8% versus 22.5±10.8, p<0.001). Aside from differences in LVEF, other baseline characteristics including age, gender, body mass index, prevalence of diabetes or arterial hypertension, history of prior myocardial infarction or stroke, previous coronary artery bypass grafting, and prevalence of atrial fibrillation were well balanced among medically treated patients. In contrast, within the TAVI cohort, patients of the T30- group substantially differed from patients of the T30+ group not only with respect to risk scores (e.g. logistic EuroSCORE 40.6±16.4% versus 22.1±12.8, p<0.001), but also with regard to baseline characteristics such as male gender (59.5% versus 41.1%, p = 0.05), previous percutaneous coronary intervention (32.4% versus 16.0%, p = 0.02), and NYHA functional class (3.0±0.6 versus 2.6±0.8, p = 0.001) (Table 1).

**Table 1:** Baseline Clinical Characteristics.

	Medical Treatment			Transcatheter Aortic Valve Implantation		
	LVEF >30%N = 46	LVEF ≤30%N = 25	P-value	LVEF >30%N = 219	LVEF ≤30%N = 37	P-value
Age (years)	83.6±6.2	82.4±5.5	0.41	82.3±6.2	81.1±6.4	0.28
Females n/%	21/45.7	9/26.0	0.46	129/58.9	15/40.5	0.05
BMI (kg/m^2^)	24.7±4.0	23.8±2.8	0.33	26.0±4.7	25.2±5.6	0.35
***Cardiac Risk Factors***						
Hypertension n/%	33/71.7	15/60.0%	0.43	172/78.5	28/75.7	0.67
Current smoker n/%	6/13.0%	0	0.08	34/15.5	7/18.9	0.63
Diabetes mellitus n/%	7/15.2	9/36.0%	0.07	49/22.4	13/35.1	0.10
Positive family history n/%	6/13.0	6/24.0	0.32	44/20.1	5/13.5	0.50
Hypercholesterolemia n/%	15/32.6	15/60.0	0.04	135/61.6	19/51.4	0.28
***Past Medical History***						
Prior MI* n/%	10/21.7	9/36.0	0.26	35/16.0	12/32.4	0.02
Prior PCI† n/%	6/13.0	14/16.0	0.73	48/21.9	10/27.0	0.53
CABG‡ n/%	8/17.4	8/32.0	0.23	44/20.1	10/27.0	0.38
Previous stroke n/%	8/17.4	4/16.0	1.0	17/7.8	6/16.2	0.12
PVD§ n/%	9/19.6	7/28.0	0.55	52/23.7	12/32.4	0.30
***Symptoms***						
NYHA || functional class	2.6±0.7	2.8±0.8	0.35	2.6±0.8	3.0±0.6	0.001
Angina n/%	19/41.3	7/28.0	0.31	65/29.7	10/27.0	0.85
Syncope n/%	10/21.7	3/12.0	0.36	20/9.1	4/10.8	0.76
***Cardiac Rhythm***						
Atrial fibrillation n/%	10/21.7	9/36.0	0.26	56/25.6	10/27.0	0.84
Prior pacemaker n/%	3/6.5	1/4.0	1.0	23/10.5	3/8.1	1.0
***Risk Assessment***						
Log. EuroSCORE^a^ (%)	22.5±10.8	38.5±13.8	<0.001	22.1±12.8	40.6±16.4	<0.001
Lin. EuroSCORE^a^ (%)	10.5±1.9	12.5±1.8	<0.001	10.3±2.2	13.1±2.3	<0.001
STS score° (%)	5.5±3.2	8.7±4.6	0.001	6.2±5.0	7.9±4.9	0.05
***Medical Treatment***						
Acetylsalicylic acid n/%	27/58.7	9/36.0	0.09	133/60.7	22/59.5	1.0
Clopidogrel n/%	8/17.4	2/8.0	0.48	37/16.9	10/27.0	0.17
Oral anticoagulation n/%	8717.4	12/48.0	0.01	61/27.9	12/32.4	0.56
Diuretic n/%	37/80.4	24/96.0	0.09	144/65.8	29/78.4	0.18
Betablocker n/%	20/43.5	9/36.0	0.62	110/50.2	23/62.2	0.21
ACE-Inhibitor/ARB n/%	21/45.7	8/32.0	0.32	94/42.9	23/62.2	0.03
Ca Channel blocker n/%	5/10.9	0	0.15	29/13.2	0	0.01
Statin (%)	15/32.6	10/40.0	0.61	106/48.4	17/45.9	0.86

*MI  =  Myocardial Infarction, †PCI  =  percutaneous coronary intervention,

‡CABG  =  Coroanry Artery Bypass Graft,

§PVD  =  Peripheral Vascular Disease, || NYHA  =  New York Heart Association (mean±standard deviation),

a EuroSCORE  =  European System for Cardiac Operative Risk Evaluation,

°STS  =  Society of Thoracic Surgeons.

**Table 2:** Imaging Characteristics.

	Medical Treatment			Transcatheter Aortic Valve Implantation		
	LVEF >30%N = 48	LVEF ≤30%N = 23	P-value	LVEF >30%N = 219	LVEF ≤30%N = 37	P-value
**Echocardiography**						
LVEF (%)	56.2±9.7	26.7±4.1	<0.001	55.6±10.0	25.2±4.4	<0.001
Mean gradient (mmHg)	44.9±15.5	35.0±21.2	0.07	45.9±16.8	35.2±15.9	0.003
AVA (cm^2^)	0.7±0.3	0.6±0.2	0.04	0.7±0.2	0.6±0.2	0.54
***Cardiac Catheterization***						
Coronary artery disease	29/63.0	15/60.0	0.80	142/64.8	25/67.6	0.85
Single-vessel CAD*	9/19.6	0	0.03	49/22.4	5/13.5	0.24
Double-vessel CAD*	8/17.4	2/8.0	0.03	27/12.3	3/8.1	0.24
Triple-vessel CAD*	12/26.1	13/52.0	0.03	66/30.1	17/45.9	0.24
Mean gradient (mmHg)	40.8±16.5	31.1±14.7	0.08	45.0±14.2	32.5±15.8	<0.001
AVA† (cm^2^)	0.6±0.2	0.7±0.3	0.27	0.5±0.2	0.6±0.2	0.75
PA‡ syst. pressure (mmHg)	56.4±20.6	59.5±16.5	0.63	52.1±16.6	62.4±15.2	0.002
* *PA‡ syst. pressure ≥ 60 mmHg	10/21.7	7/28.0	0.57	45/20.5	16/43.2	0.006

*CAD  =  Coronary Artery Disease,

†AVA  =  Aortic Valve Area,

‡PA  =  Pulmonary Artery.

Mean LVEF amounted to 56.2±9.7% (M30+) and 26.7±4.1% (M30-) in patients assigned to a medical strategy, and to 55.6±10.0% (T30+) and 25.2±4.4% (T30-) in patients undergoing TAVI. While mean gradients were lower in patients with LVEF≤30% among TAVI patients (32.5±15.8 mmHg for T30- versus 45.0±14.2 mmHg for T30+, p<0.001), the numerical difference in the medical cohort fell short of statistical significance (35.0±21.2 mmHg for M30- versus 44.9±15.5 mmHg for M30+, p = 0.07). Among T30- the prevalence of patients with a mean gradient ≤30 mmHg amounted to 45.9%. Furthermore, LVEF ≤30% in patients undergoing TAVI went along with a higher rate of severe pulmonary hypertension (62.4±15.2 mmHg for T30- versus 51.1±16.6 mmHg for T30+, p = 0.006). In contrast, patients with LVEF≤30% in the medical cohort had a higher prevalence of triple vessel coronary artery disease (52.0% for M30- versus 26.1% for M30+, p = 0.03) (Table 2).

### Short-term Clinical Outcomes

**Figure 2:**
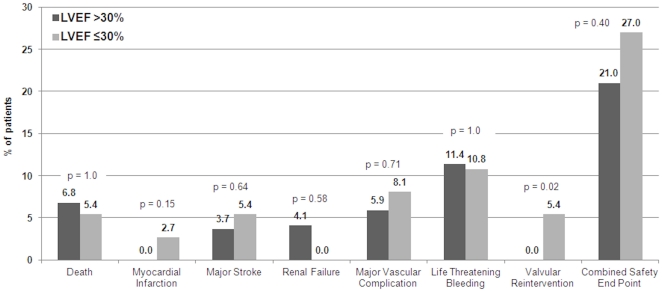
Short-term clinical outcome as assessed by the Valve Academic research Consortium Combined Safety Endpoint at 30 days in patients undergoing TAVI stratified by LVEF>30% or ≤**30%.**

Peri-procedural characteristics and short-term clinical outcome of patients undergoing TAVI are summarized in Tables 3 and 4. All-cause mortality at 30 days amounted to 6.8% and 5.4% among patients of the T30+ and T30- group (p = 1.0), respectively. There were no differences between patients of group T30+ and T30- with regard to peri-procedural myocardial infarction (0% versus 2.7%, p = 0.15), major stroke (3.7% versus 5.4%, p = 0.64), access related complications, bleeding and renal failure. Two patients of the group T30- required valvular reinterventions, whereas no valvular reinterventions were performed among patients of the group T30+ (5.4% for T30- versus 0% for T30+, p = 0.02). Both patients underwent post-dilatation 13 days and 14 days after TAVI, respectively, due to severe aortic regurgitation. There was no significant difference in the incidence of the Valve Academic Research Consortium (VARC) combined safety end point [11] between the two groups (21.0% for T30+ versus 27.0% for T30-, p = 0.40) (Figure 2). Aortic regurgitation >grade 2+ as assessed by transthoracic echocardiography before discharge was found in 26.6% of patients with T>30% versus 36.1% in patients T≤30% (p = 0.31).

**Table 3:** Procedural Characteristics.

	Medical Treatment			Transcatheter Aortic Valve Implantation		
	LVEF >30%N = 48	LVEF ≤30%N = 23	P-value	LVEF >30%N = 219	LVEF ≤30%N = 37	P-value
**Aortic Valve Implantation**						
Transfemoral MCV*n/%	na	na	na	137/62.6	23/62.2	0.86
Transsubclavian MCV*n/%	na	na	na	4/1.8	0	0.86
Transfemoral ES† n/%	na	na	na	31/14.2	6/16.2	0.86
Transapical ES† n/%	na	na	na	47/21.5	8/21.6	0.86
***Revascularization***						
Concomitant PCI‡ n/%	7/15.2%	3/12.0%	1.0	23/15.5	3/8.1	0.32
Staged PCI‡ n/%	na	na	na	21/9.6	2/5.4	0.55
***Hospitalization Duration***	4.6±3.7	4.4±3.7	0.84	10.7±5.4	12.3±9.2	0.13

*MCV  =  Medtronic CoreValve biosprosthesis,

†ES  =  Edwards Sapien bioprosthesis,

‡PCI  =  Percutaneous Coronary Intervention.

**Table 4:** Outcome at 30 Days According to Systolic Left-Ventricular Function.

	Transcatheter Aortic Valve Implantation		
	LVEF >30%N = 219	LVEF ≤30%N = 37	P-value
***All Cause Mortality n/%***	15/6.8	2/5.4	1.0
Cardiovascular mortality n/%	10/4.6	2/5.4	0.69
***Myocardial Infarction n/%***	0	1/2.7	0.15
***Major Stroke n/%***	8/3.7	2/5.4	0.64
***Access related complications n/%***			
Major n/%	13/5.9	3/8.1	0.71
Minor n/%	19/8.7	4/10.8	0.76
***Valvular Reintervention n/%***	0	2/5.4	0.02
***Bleeding n/%***			
Life-threatening n/%	25/11.4	4/10.8	1.0
Major n/%	68/31.1	10/27.0	0.70
***Renal complications n/%***			
RIFLE* Stage 1 n/%	27/12.3	5/13.5	0.58
RIFLE* Stage 2 n/%	2/0.90	0	0.58
RIFLE* Stage 3 n/%	9/4.1	0	0.58
**Pacemaker Implantation ** ***n/%***	48/21.9	12/32.4	0.21
***VARC*** † ***-Combined Safety End Point n/%***	46/21.0	10/27.0	0.40

*RIFLE  =  Risk, Injury, Failure, Loss, End-stage kidney disease,

†VARC  =  Valve Academic Research Consortium.

**Figure 3:**
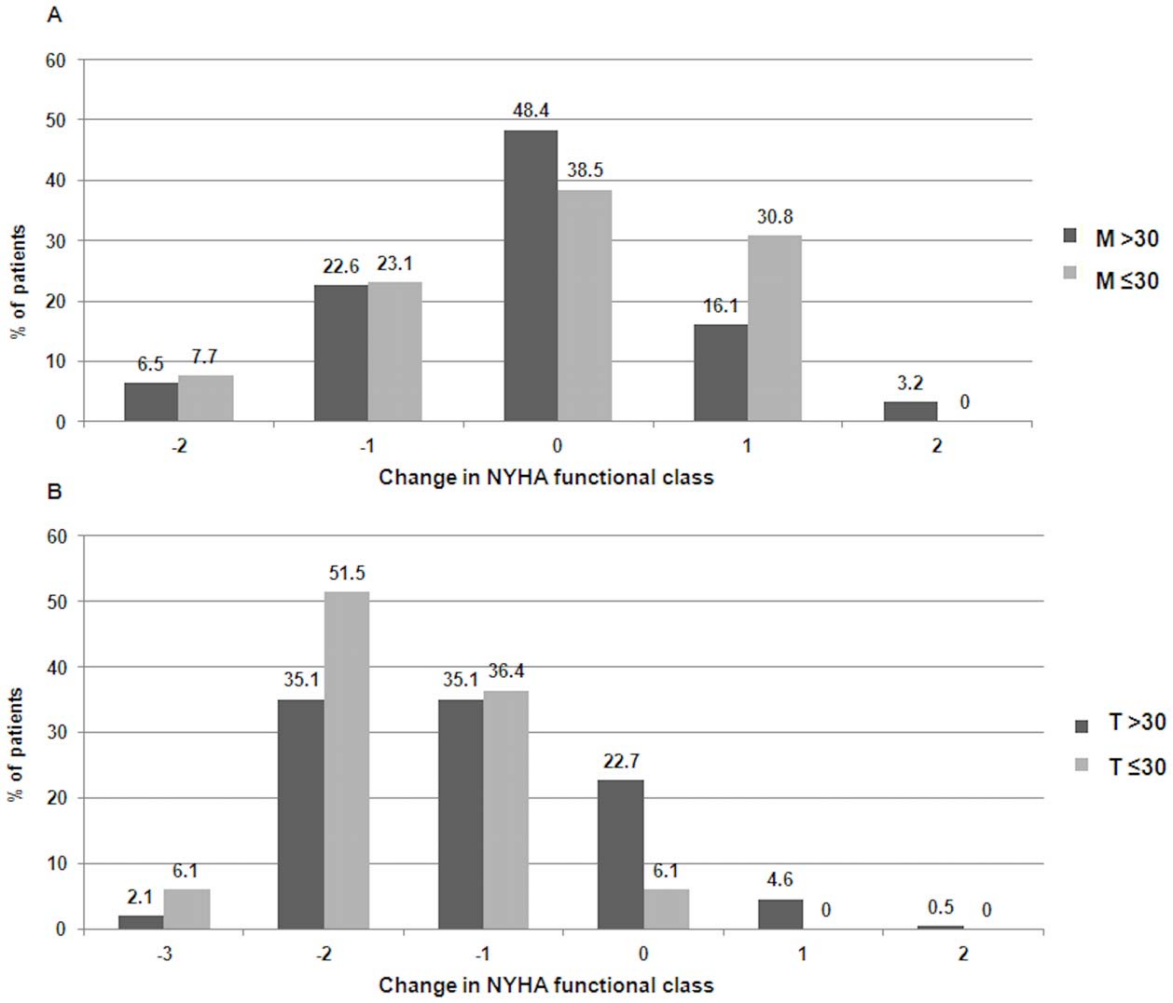
Changes in NYHA Functional Class at 30 days in patients undergoing medical treatment (A) or transcatheter aortic valve implantation (B) according to LVEF >30% or ≤30%.

Exercise intolerance as assessed by NYHA functional class was documented at 30 days and is illustrated in Figure 3. Whereas patients under medical treatment reported an increase in shortness of breath at 30 days (increase ≥1 NYHA class in 30.8% of M30- and 19.3% of M30+), TAVI patients consistently noted an improvement in exercise tolerance that seemed to be particularly pronounced among patients of the T30- group (decrease ≥1 NYHA class in 95% of T30- and 77.3% of T30+).

### Long-term Follow-Up

**Figure 4:**
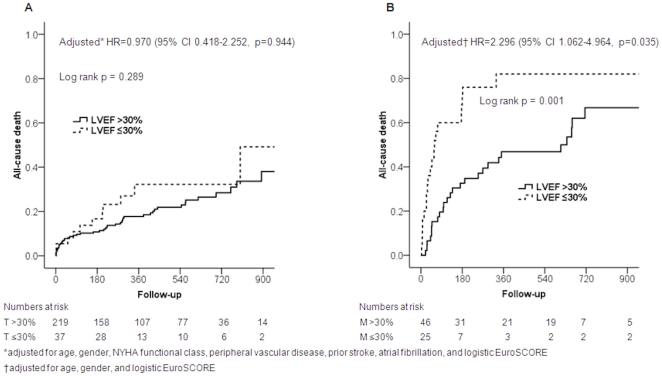
Survival in patients undergoing medical treatment or TAVI as a function of LVEF.

Crude and adjusted long-term survival of patients undergoing TAVI and medical treatment as a function of LVEF is shown in Figure 4. Patients undergoing TAVI experienced a favorable long-term course compared with patients under medical treatment. There was no difference in survival between patients of the T30- and T30+ group during long-term follow-up in a crude analysis, as well as after adjustment for age, gender, NYHA functional class, peripheral vascular disease, prior stroke, atrial fibrillation, and logistic EuroSCORE (HR 0.97, 95% CI 0.42-2.25; p = 0.94). Conversely, patients of the M30- group had a significantly higher mortality exceeding 80% at one year as compared with patients of the M30+ group (p = 0.001). This difference was maintained after adjustment for age, gender, and logistic EuroSCORE (HR 2.30, 95% CI 1.06-4.96; p = 0.04).

**Figure 5:**
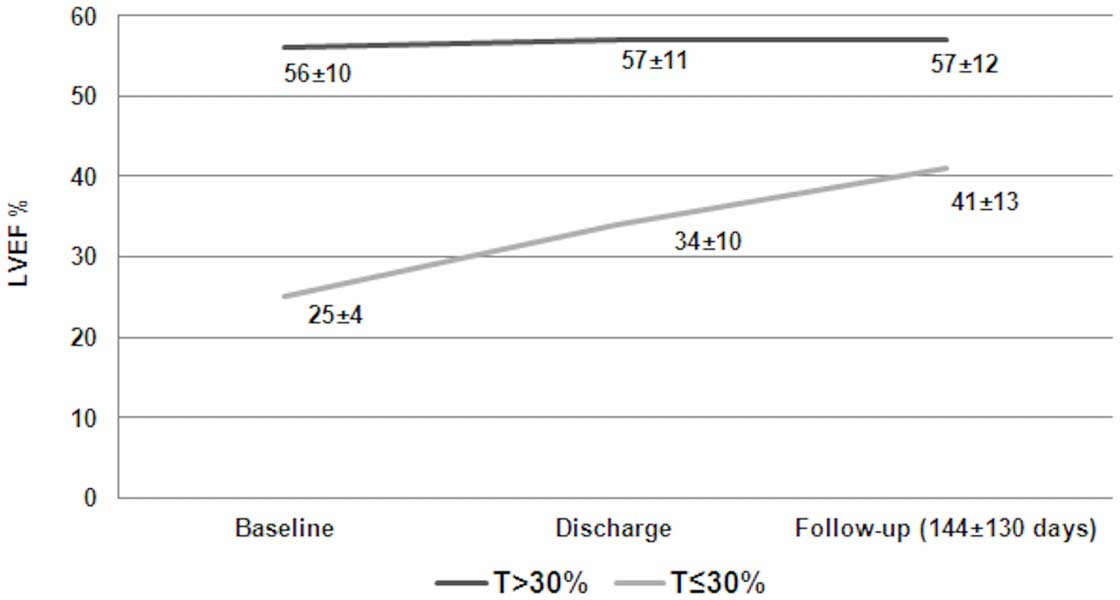
LVEF on admission, improvement during the in-hospital phase, and after a mean follow-up duration of 144±130 days.

Echocardiographic follow-up was available in 93% of patients at discharge and in 79% at a mean follow-up of 144±130 days. No echocardiographic follow-up was performed in patients allocated to medical treatment. Patients of the T30- group experienced recovery of LVEF from baseline to discharge (from 25±4% to 34±10%, p = 0.002) that continued to increase during mid-term follow-up (41±13%) (Figure 5).

## Discussion

### The main findings of the study are as follows

1. Severely diminished left ventricular function among patients with severe aortic stenosis treated medically has an important impact on clinical outcome with dismal prognosis.
2. TAVI in patients with severely impaired left ventricular function may be performed safely and may not be associated with an increased peri-procedural risk.
3. Patients with severely diminished LVEF undergoing TAVI demonstrate a rapid improvement in LV function and may have a similar prognosis as compared to patients with normal or moderately reduced LVEF.
4. Among patients with severely diminished LVEF, TAVI disproportionally improves NYHA functional class as compared with patients with normal or moderately reduced LVEF.

The observational data of the present single center experience demonstrate the favorable impact of TAVI on recovery of LVEF and long-term clinical outcome as compared with medical treatment. Moreover, the risk of peri-procedural complications in patients with severely impaired LVEF appears to be comparable to patients with normal or mildly reduced LVEF.

There are several limitations to be considered. First, the observational, non-randomized nature of the present analysis is susceptible to a selection bias attributable to the primary allocation of the selected treatment strategy. The decision whether to perform TAVI or medical treatment in patients with severely impaired LVEF was driven by the decision of the interdisciplinary heart team, by the feasibility based on anatomic and technical features, and by the final decision of the patient. Second, the threshold values for categorization of the overall patient cohort according to LVEF, although arbitrary, were based on previous clinical studies of patients undergoing SAVR indicating an association of impaired LVEF with adverse clinical outcome [1]–[5]. At last, we might have been unaware of unknown confounding factors influencing clinical outcome.

Our data highlight the dismal prognosis of patients with severe, symptomatic aortic stenosis undergoing medical treatment. The overall estimated rate of mortality after one year amounted to 55% and is in line with the results of the medical group of the PARTNER B cohort (Placement of AoRtic TraNscathetER Valve) trial [7]. In our series, stratification according to LVEF demonstrates comparable baseline characteristics but an excessive risk of mortality among patients with severely reduced LVEF.

The assessment of peri-procedural complications in the TAVI cohort using the VARC criteria revealed no differences between the two groups stratified according to LVEF. In particular, we observed no differences with regard to all-cause mortality, incidence of peri-procedural myocardial infarction or major stroke. The most frequently encountered peri-procedural complications were bleeding events and vascular complications, which occurred with similar frequency in both groups. A higher rate of repeat valvular interventions among patients of the T30- group may be due to chance but needs further investigation.

The overall incidence of adverse events was comparable with previous reports of patients undergoing TAVI [12]–[14] and suggests that TAVI in patients with severely reduced left ventricular function is not associated with an increased peri-operative risk. This finding contrasts with data from the surgical literature showing an increased risk of adverse events of patients with reduced LVEF [1]–[5]. For instance, Sharony et al reported a 30-day mortality of 9.6% among patients with LVEF≤40% in a series of 260 patients [3]. Several factors might explain the negligible role of a diminished LVEF during the peri-procedural phase of TAVI: the strategy of a pure percutaneous approach using local anesthesia and mild conscious sedation reduces the risk of unfavorable hemodynamics during the intervention and the need of vasoactive drugs. Positioning of the stiff wire in the left ventricle and the deployment of the bioprosthesis is considered to be easier in patients with an enlarged ventricle with low output compared with patients with a small, hypertrophic and hypercontractile ventricle. Furthermore, TAVI provides the possibility of valve implantation without cardiac arrest and its sequelae like the need for prolonged ventilation, the risk of renal failure, infection and neurologic complications [15].

Our data show, that the combination of severe aortic stenosis and severely reduced left ventricular function is associated with a dismal prognosis if treated conservatively. In our cohort, more than half of the patients died within six months of evaluation for potential intervention and almost 80% died within one year. The present findings therefore suggest that severely impaired left ventricular function should not serve as a reason to deny transcatheter aortic valve implantation. The favorable peri-procedural outcome was accompanied by a rapid recovery in LVEF already during the in-hospital phase and eventually translated into favorable long-term survival comparable to patients with normal or moderately reduced LVEF. Of note, the mean aortic transvalvular gradient in patients with LVEF≤30 amounted to 35±16 mmHg indicating that low-flow, low-gradient aortic stenosis was encountered relatively infrequently and a majority of patients may have maintained some contractile function. Since we did not routinely perform dobutamine stress echocardiography the issue whether contractile reserve plays an important role with respect to prognosis remains unanswered. Nevertheless, patients with LVEF≤30% assigned to medical treatment exhibited similar transvalvular gradients (35±21 mmHg) and were found to have a considerably higher mortality rate as compared to medically treated patients with LVEF>30%.

Among patients with severely impaired LVEF symptom status as assessed by NYHA functional class improved disproportionally along with a rapid recovery in systolic left ventricular function. These findings suggest that patients with severely reduced LVEF may substantially benefit from TAVI.

### Conclusions

TAVI in patients with severely reduced left ventricular function may be performed safely and is associated with rapid recovery of systolic left ventricular function and heart failure symptoms.
